# Proof of concept: Comparative accuracy of semiautomated VR modeling for volumetric analysis of the heart ventricles

**DOI:** 10.1016/j.heliyon.2022.e11250

**Published:** 2022-11-01

**Authors:** David Yogev, Shai Tejman-Yarden, Omer Feinberg, Yisrael Parmet, Tomer Goldberg, Shay Illouz, Netanel Nagar, Dor Freidin, Oliana Vazgovsky, Sumit Chatterji, Yishay Salem, Uriel Katz, Orly Goitein

**Affiliations:** a The Sackler School of Medicine, Tel Aviv University, Tel Aviv, Israel; b The Engineering Medical Research Lab, Sheba Medical Center, Ramat Gan, Israel; c The Edmond J. Safra International Congenital Heart Center, Sheba Medical Center, Ramat Gan, Israel; d Department of Industrial Engineering and Management, Ben Gurion University, Beer Sheva, Israel; e Industrial Design Department, Bezalel Academy of Art and Design, Jerusalem, Israel; f The Leviev Heart Institute, Sheba Medical Center, Ramat Gan, Israel; g The Pulmonology Unit, Sheba Medical Center, Ramat Gan, Israel; h Interventional Pulmonology Unit, Sheba Medical Center, Ramat Gan, Israel; i Department of Diagnostic Imaging, Sheba Medical Center, Ramat Gan, Israel

**Keywords:** Virtual reality, Polygon summation, Volumetric analysis, Heart chambers

## Abstract

**Introduction:**

Simpson's rule is generally used to estimate cardiac volumes. By contrast, modern methods such as Virtual Reality (VR) utilize mesh modeling to present the object's surface spatial structure, thus enabling intricate volumetric calculations. In this study, two types of semiautomated VR models for cardiac volumetric analysis were compared to the standard Philips dedicated cardiac imaging platform (PDP) which is based on Simpson's rule calculations.

**Methods:**

This retrospective report examined the cardiac computed tomography angiography (CCTA) of twenty patients with atrial fibrillation obtained prior to a left atrial appendage occlusion procedure. We employed two VR models to evaluate each CCTA and compared them to the PDP: a VR model with Philips-similar segmentations (VR-PS) that included the trabeculae and the papillary muscles within the luminal volume, and a VR model that only included the inner blood pool (VR-IBP).

**Results:**

Comparison of the VR-PS and the PDP left ventricle (LV) volumes demonstrated excellent correlation with a $\rho_{c}$ of 0.983 (95% CI 0.96, 0.99), and a small mean difference and range. The calculated volumes of the right ventricle (RV) had a somewhat lower correlation of 0.89 (95% CI 0.781, 0.95), a small mean difference, and a broader range. The VR-IBP chamber size estimations were significantly smaller than the estimates based on the PDP.

**Discussion:**

Simpson's rule and polygon summation algorithms produce similar results in normal morphological LVs. However, this correlation failed to emerge when applied to RVs and irregular chambers.

**Conclusions:**

The findings suggest that the polygon summation method is preferable for RV and irregular LV volume and function calculations.

## Graphical abstract

**Figure:**
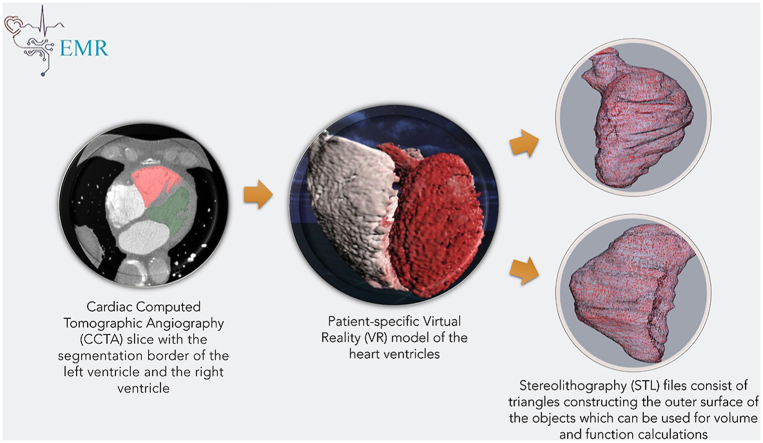

## 1 Introduction

Transthoracic echocardiography (TTE), cardiac computed tomography angiography (CCTA), and cardiac magnetic resonance (CMR) are routinely performed to assess the size and function of the cardiac chambers [1, 2, 3, 4]. While CMR is considered the gold standard for cardiac chamber volume measurements [5], CCTA has proven to be equally reliable and accurate, and both are routinely used in clinical settings [3, 4, 6]. TTE is widely available and relatively less expensive. Generally, TTE is used for anatomical and left ventricular (LV) function assessments even though it is less accurate, due to its low spatial resolution and high intra-observer variability [2, 7, 8].

All three modalities use Simpson's rule to calculate the cardiac chambers' end-diastolic volumes (EDV) and end-systolic volumes (ESV) [3, 4, 7]. The chambers' endocardial borders are marked on each slice, the luminal area is multiplied by the slice thickness, and the volumes of the slices are then added to obtain the chambers' volumes during the cardiac cycle [3, 7]. Standardized evaluations include the ventricles, without the outflow tracts. The papillary muscles and the trabeculae are included within the luminal volume, creating smoothed ventricular contours. In the LV, calculations are performed in the short axis plane according to standardized contouring, where basal slices with a semicircular muscular ring of less than 50% of the LV circumference at end-systole are disregarded [3, 4]. End-diastole and end-systole are detected manually for TTE and automatically for CCTA and CMR according to the largest and the smallest ventricular volume for EDV and ESV evaluations, respectively. These volumetric assessments are then used for ventricular stroke volume and ejection fraction calculations [3, 5].

Virtual reality (VR) imaging may be a viable alternative for cardiac volumetric analysis. It allows for comprehensive visualization of complex anatomical structures such as those present in congenital heart defects and may contribute to perioperative planning and interventions [9, 10, 11, 12]. VR three-dimensional (3D) spatial imaging uses semiautomated tools that combine automatic segmentation with operator supervision. The segmentation is based on tissue enhancement that enables an accurate separation of the different structural components and makes it possible to identify the heart's inner blood pool, the myocardial tissue, and the surrounding mediastinal organs [12, 13]. Unlike other imaging methodologies, the VR modeling language (VRML) applies mesh models (layer models) that utilize a set of 3D geometric entities, including vertices or polygons to represent the detailed surfaces of spatial objects [14]. Features such as volume measurements, intricate inter-organ relations, and tissue biomechanical properties can be extracted from the mesh representations efficiently and accurately [13, 14, 15, 16, 17, 18, 19].

Compared to other methods, semiautomated VR segmentation is quick, accurate, and less prone to inter-observer variability. Thus, this methodology may facilitate additional assessments in complex anatomical environments [16, 19]. The use of 3D mesh model analysis has been studied in both cardiology and other fields such as orthopedics, dentistry, and others [20, 21, 22]. The current study was designed to examine the feasibility of semiautomated VR volumetric analysis for the accurate measurement of cardiac chamber volumes.

## 2 Methods

### 2.1 Patients and image collection

This retrospective study was conducted in a single center. The dataset was derived from the records of adult patients followed at the Sheba Medical Center (SMC) Heart Institute between 2017 and 2019. All the patients in the cohort underwent a dedicated CCTA scan. The study protocol was approved by the SMC institutional review board. The participants’ informed consent was waived since the data were retrieved without identificatory information from medical records. Data treatment adhered to the principles of Good Clinical Practice (GCP).

### 2.2 Image analysis

CCTA was performed using retrospective gating utilizing a 256-slice scanner (Brilliance iCT; Philips Healthcare, Cleveland, OH, USA) with 70 ml of intravenous non-ionic contrast medium (Iomeron 350, Bracco, Milano, Italy) followed by 40 ml of saline flush (at an injection rate of 4–5 ml/s). The CCTA data were reconstructed using a dedicated platform (Comprehensive Cardiac Analysis, Extended Brilliance Workspace (version 4.5); Philips Healthcare). Volumetric analysis was conducted using the best available diastolic phase that represented 80%–90% of the R-R interval.

The VR simulated heart models were created retrospectively based on the same CCTA data collected for each patient. Images were uploaded as Digital Imaging and Communications in Medicine (DICOM) files into D2P® software (3D Systems Inc. Littleton CO, USA) for cardiac chamber segmentation. The mesh files were converted into stereolithography (STL) format files that describe 3D objects’ surface geometry. The operator was able to see the resulting segmentation in a stereoscopic view using a dedicated system (Vive System, HTC, San Francisco, CA, USA).

### 2.3 Volumetric chamber analysis

**Figure 1:**
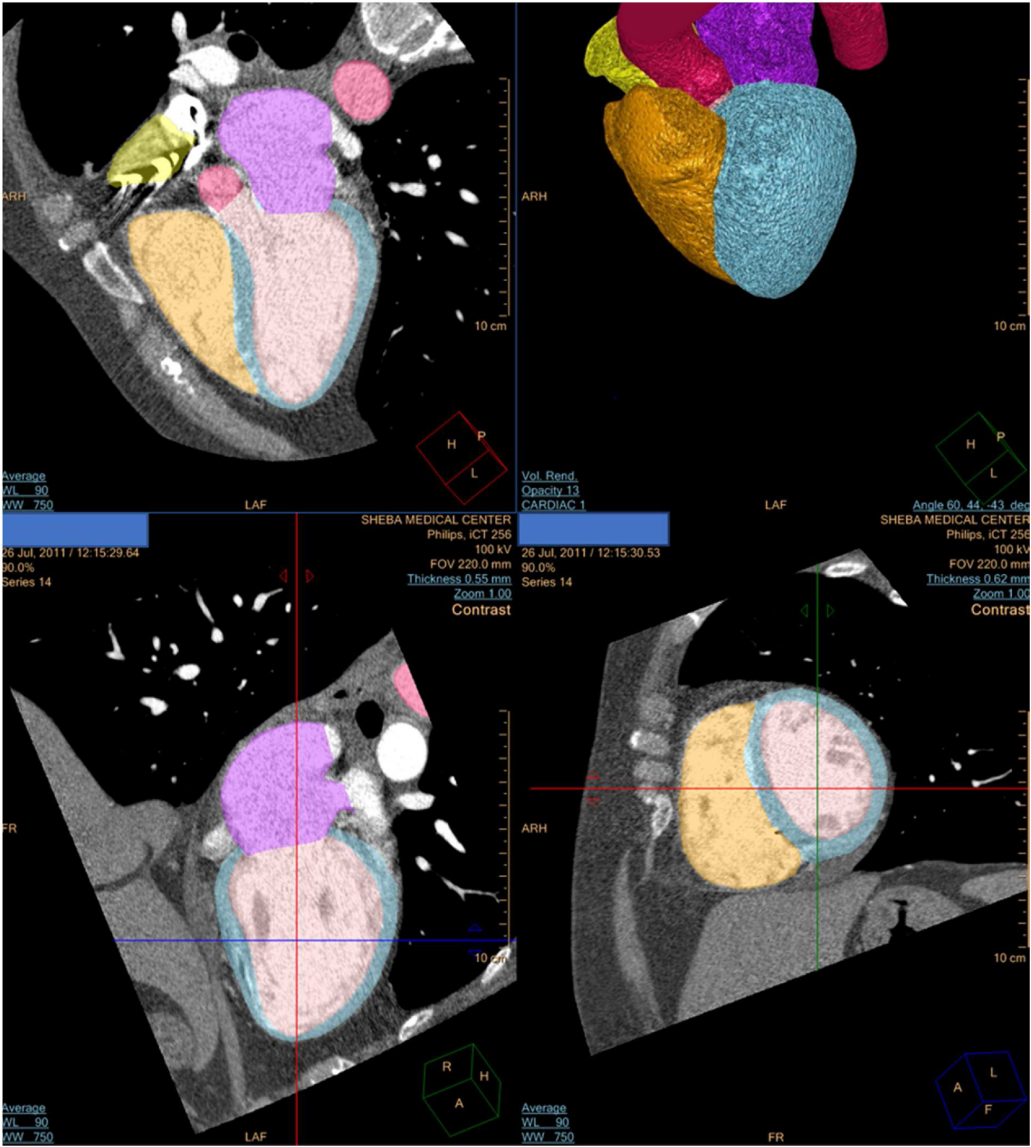
The Cardiac Computed Tomographic Angiography (CCTA) data automatic reconstruction using a dedicated cardiac imaging platform (Comprehensive Cardiac Analysis, Extended Brilliance Workspace (version 4.5); Philips Healthcare).

**Figure 2:**
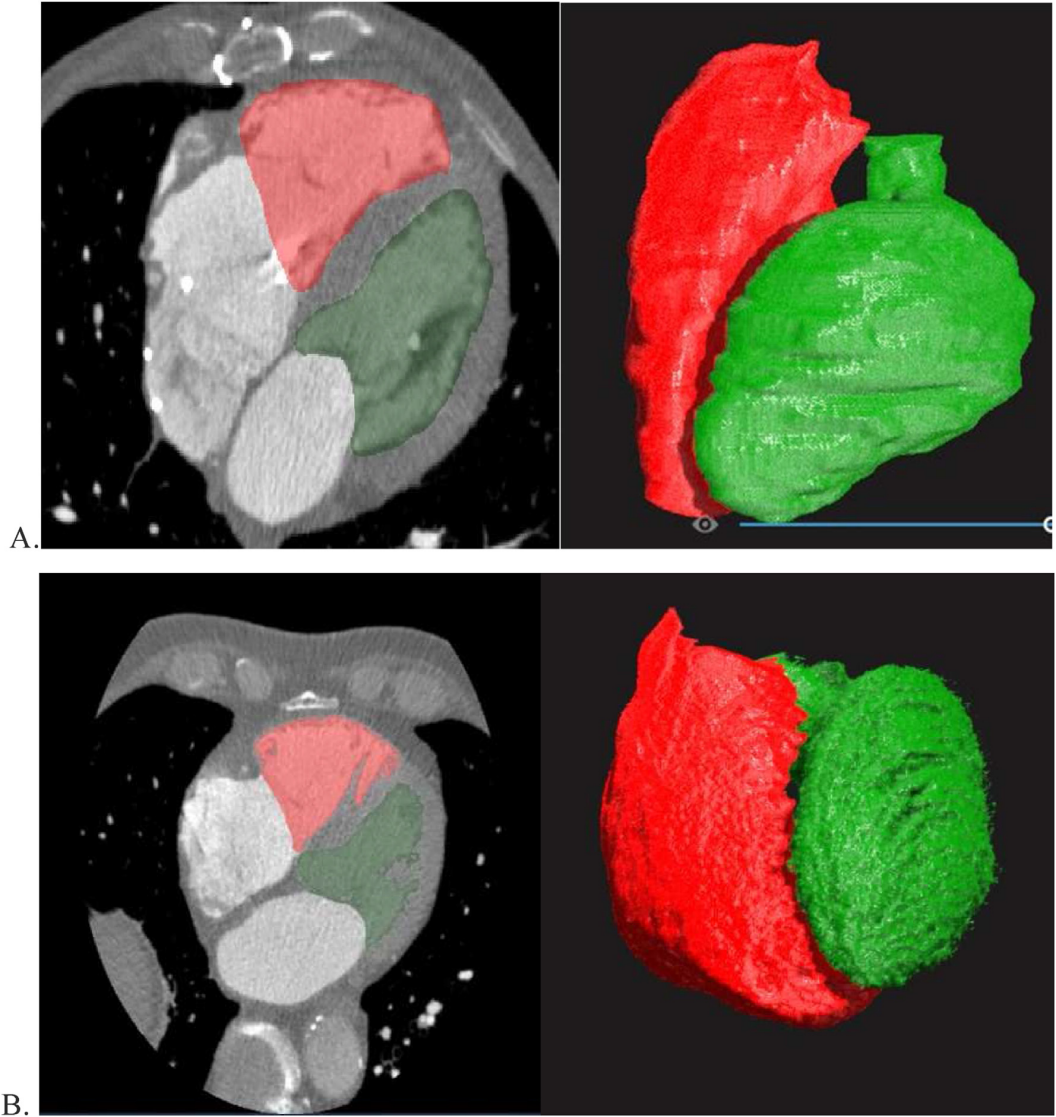
Virtual Reality (VR), three-dimensional imaging, and segmentation including the trabeculae and the papillary muscles produced a smooth border similar to the Philips platform segmentation. A - Cardiac Computed Tomographic Angiography (CCTA) slice and a 3D model with the segmentation border of the left ventricle and the right ventricle, including the trabeculae and the papillary muscles. B - VR imaging and segmentation of the inner blood pool inside the heart chambers.

For the volumetric analysis, each heart was evaluated in the same phase using three different analysis methods. The first implemented the Phillips dedicated cardiac CT imaging platform (PDP) (Figure 1), and the other two used the D2P® segmentation software (Figure 2) for volumetric analysis.

### 2.4 Volume calculation based on Simpson's rule

The Philips software automatically reconstructed the analyzed ventricles, and their volumes were measured. The dedicated cardiac CT imaging platform segmented the inner ventricular area. The measurements included the ventricular trabeculae and both chambers' papillary muscles, as is common practice (Figure 1). The base of the trabeculae adjacent to the inner myocardium was defined as the edge of the segmentation. The volumetric mathematical analysis in the Philips' software is based on Simpson's rule of disk-area summation [9]. The chamber was divided into defined, equally separated slices. The voxel area of each slice was measured and then multiplied by the defined slice thickness to create a 3D phantom of the measured cavity. The 3D geometric shape of the chamber was smoothed so that it approximated the conventional bullet shape in the LV and followed the tri-partite structure in the right ventricle (RV).

### 2.5 Volume calculation based on the ventricular surface

Segmentations based on the outer surfaces of the two ventricles were performed using the D2P® segmentation software. The tissue segmentations were done semi-automatically with two different defined segmentation borders. The first segmentation was the Philips-similar VR segmentation (VR-PS), which included the trabeculae and the papillary muscles within the luminal volume, which produced a smooth edge similar to the Philips platform segmentation (Figure 2A). The second segmentation only included the inner blood pool within the chamber cavity and excluded the trabeculae and the papillary muscles. The inner blood pool VR segmentation (VR-IBP) was conducted in a semiautomated manner. The operator needed to choose the appropriate Hounsfield unit range for each segmented contour, which thus precisely defined the endocardium's border enveloping the inner blood pool without smoothing or any approximation (Figure 2B).

**Figure 3:**
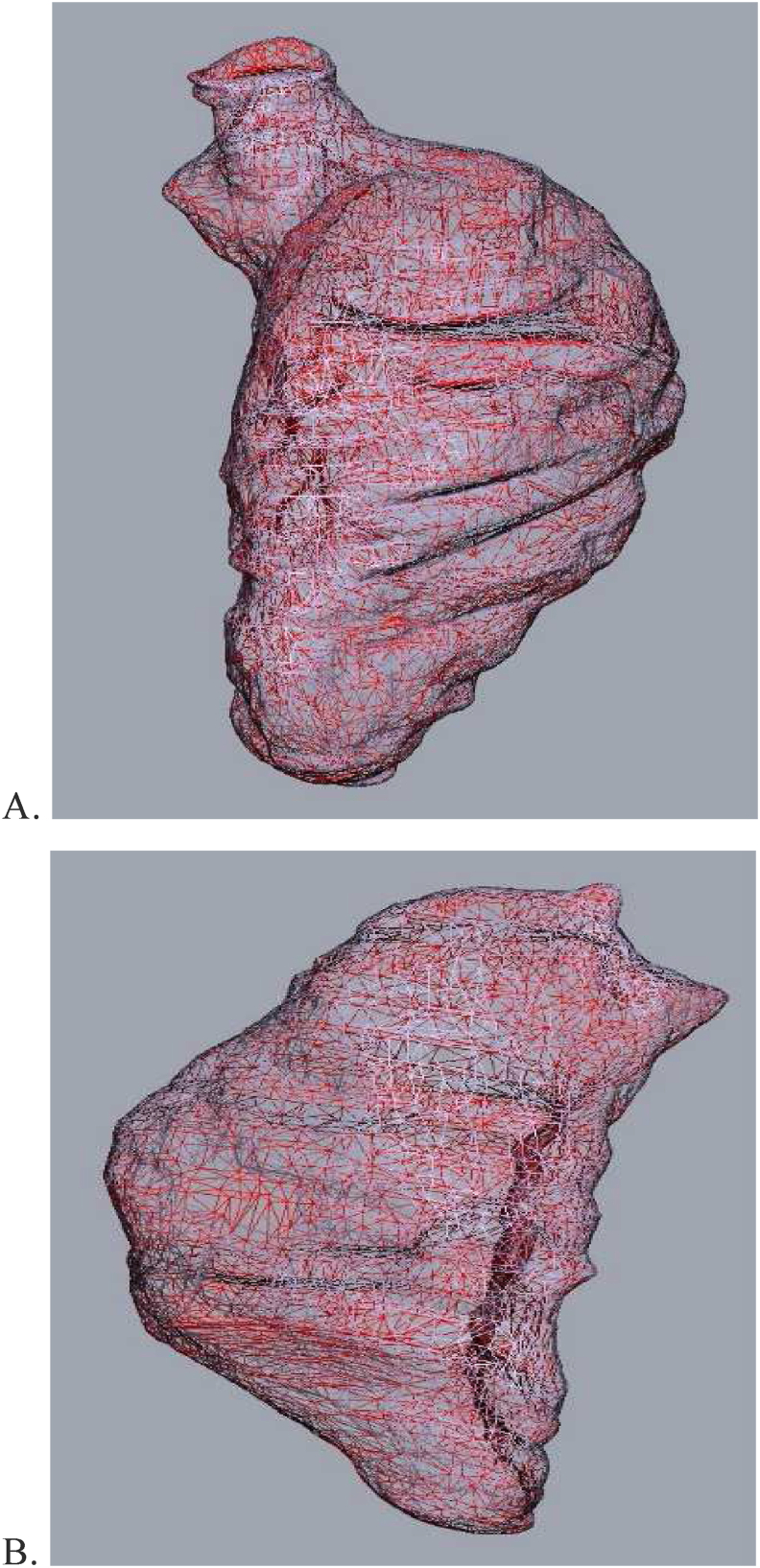
Stereolithography (STL) files consist of triangles constructing the outer surface of the objects. A – An STL file of the left ventricle. B – An STL file of the right ventricle.

In practice, when using the D2P® segmentation, a 3D mesh is created of the cavity's outer surface consisting of triangular and polygonal elements that create a phantom of the chosen ventricle (Figures 3A and 3B). This mesh is stored as an STL file. Using a dedicated Phyton® script based on established and validated mesh modeling software [19], the volume of the chambers are accurately calculated based on summation of the spatial geometric structures. The ventricular volume itself has an irregular geometry and is composed of many convex entities that can be represented in tetrahedron form. By dissecting this large structure into concave portions and summing the minute volumes, the entire volume can be accurately represented, which may ultimately be as convex or concave as the LV and RV. In terms of mathematics, the STL file contains a list of triangles forming a tetrahedron with the origin. The volume of all tetrahedrons is summed, while triangles of which the normal vector points away from the origin are accounted for as having positive volumes, and those of which the normal vector points towards the origin are accounted for as having negative volumes. The volume of each tetrahedron is given by the equation $\frac{1}{3}a*h$, where *a* is the area of the triangle, and h is its height. Denoting the locations of the triangle vertices as $p_{i,j}$, where i is the triangle index and j∈(1,2,3), and using vector calculus, the same calculation can be converted directly into the more useable expression $V=\frac{1}{6}\vec{p_{1}}\times \vec{p_{2}}\cdot \vec{p_{3}}$. With this given, the entire ventricular volume can be formulated by Eq. (1):

(1) $$V=\frac{1}{6}\sum_{i}\vec{p_{i,1}}\times \vec{p_{i,2}}\cdot \vec{p_{i,3}}$$

Note that the order of vertices matters here, as it yields the positivity or negativity of each part of the summation.

### 2.6 Statistical analysis

We examined the correlations between the VR chamber's volumetric measurements and the Phillips dedicated cardiac CT imaging platform (PDP), which is considered the gold standard for volumetric measurements. The concordance between the variables was evaluated using Lin's Concordance Correlation Coefficient ($\rho_{c}$), which combines measures of both precision and accuracy, and whose values range from -1 to 1, with perfect agreement at 1. To evaluate the differences between methods, the mean difference, the standard deviation and the mean squared error were calculated (MSE) [23, 24, 25]. The equivalence of measures was subjected to the Bland-Altman (B&A) method, and graphically presented as B&A plots, using R4.0.3 statistical analysis software [26, 27]. A paired t-test was applied to examine the correlation between two continuous data groups, such as the measurements obtained with the different VR volumetric analysis-based methods. All statistical tests were two-tailed. The Shapiro-Wilk test was applied to test the normality of the continuous data. The data are expressed as the mean ± the standard deviation (SDs) for normally distributed variables.

## 3 Results

The database was composed of the records of 20 adult patients (13 males and 7 females) with a mean age of 83.4 years (±13.0 years). Their demographics and associated comorbidities are presented in Table 1. The patients had undergone a CCTA scan as part of their evaluation before a left atrial appendage occlusion procedure due to atrial fibrillation (AF), according to the standard guidelines. For each patient, both ventricles were evaluated separately, using the three different methods: the Phillips dedicated cardiac imaging platform (PDP), a VR analysis with a Philips-similar VR segmentation (VR-PS), and a VR analysis with an internal blood pool-only segmentation (VR-IBP). The PDP measurements are the standard practice and served as the standard of reference for the VR measurements. Table 2 lists the measurements obtained by each method used to evaluate the ventricular volumes.

**Table 1:** Demographics and Associated Comorbidities in 20 patients evaluated by CT, 2017–2019.

Variables	Sample (n = 20)
Sex, n (%)	
Males	13 (65)
Females	7 (35)
Age (mean, SD)	83.4 ± 13.0
Associated comorbidities, n (%)	
Atrial fibrillation	20 (100)
Hypertension	16 (80)
Dyslipidemia	10 (50)
Ischemic heart disease	9 (45)
Type II diabetes	7 (35)
Heart failure	5 (25)
History of smoking	4 (20)
Hypothyroidism	4 (20)
History of cerebrovascular accident	4 (20)

Abbreviations: CT, computed tomography; SD, standard deviation.

**Table 2:** Volumetric Analysis of the Right and Left Ventricles measured by the three different methods.

Measurement Method	LV volume (n = 20)		RV volume (n = 20)	
	Mean ± SD (cm^3^)	Range (cm^3^)	Mean ± SD (cm^3^)	Range (cm^3^)
Phillips dedicated CCTA platform	140.4 ± 38.1	81.0–223.7	169.7 ± 32.8	101.0–237.0
Philips-similar VR segmentation	140.2 ± 40.1	81.9–229.5	164.4 ± 45.1	78.9–255.4
Inner-blood pool VR segmentation	121.0 ± 36.2	71.8–205.1	155.5 ± 39.4	92.0–248.9

The data are expressed as the mean ± standard deviation (SD). **Abbreviations:** LV = left ventricle; RV = right ventricle; CCTA = cardiac computed tomographic angiography; GS = gold standard; VR = virtual reality.

**Figure 4:**
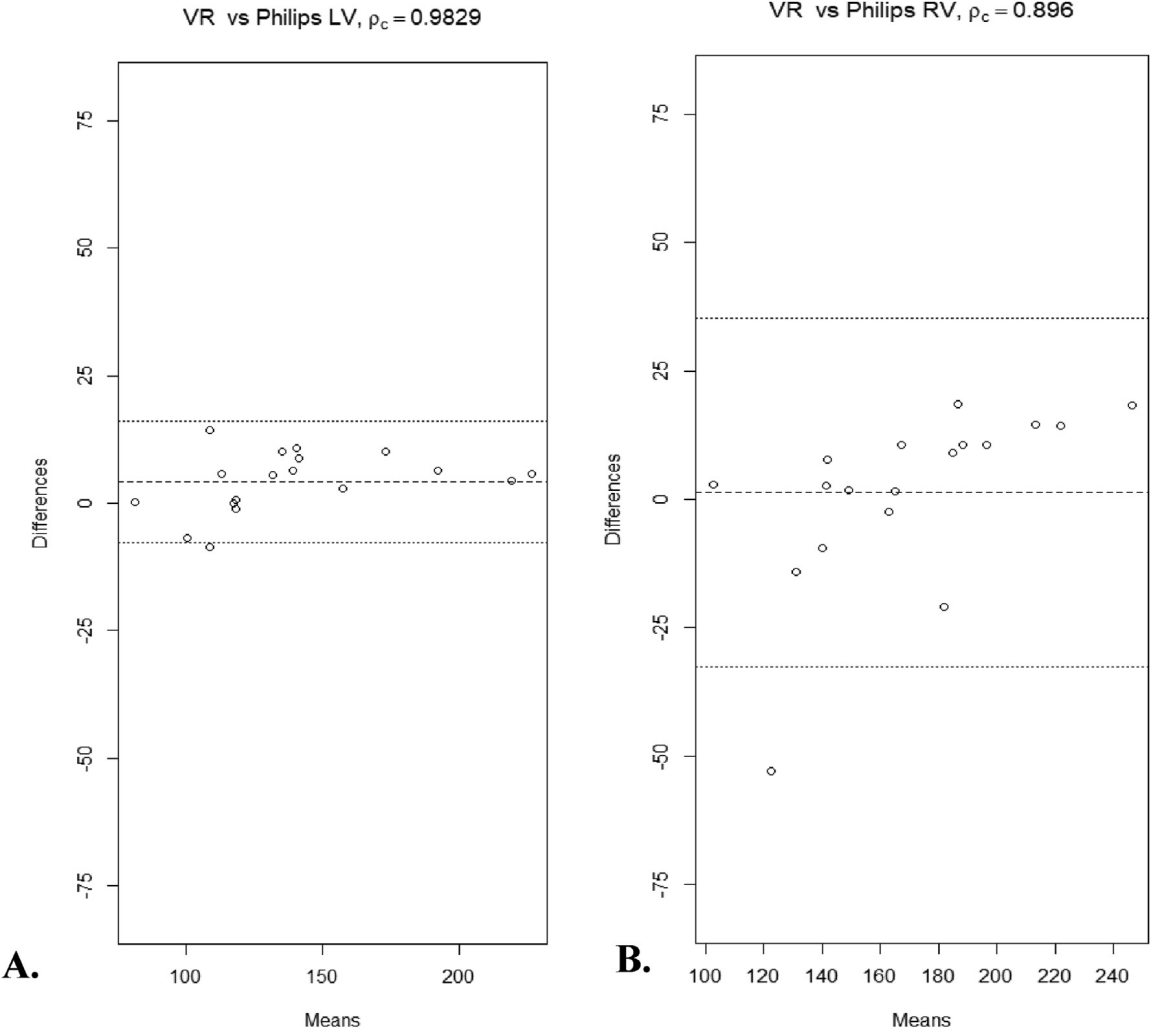
Bland-Altman plots assessing measurement equivalence between the Virtual Reality model with Philips-similar segmentation (VR-PS) measurements and the standard Philips dedicated cardiac imaging platform (PDP), for the left ventricle (LV, A), and the right ventricle (RV, B). Abbreviations: $\rho_{c}$ = Lin's Concordance Correlation Coefficient.

The Lin's Concordance Correlation Coefficient (ρ_c_) between the VR-PS and the PDP LV volume measurements was 0.983 (95% CI 0.96, 0.99). The B&A plots showed very good agreement, with a mean difference of 4.19 mm^3^ ± 6.04 (95% CI –7.65, 16.05) and a MSE of only 52.16 mm^6^ (Figure 4A). The ρ_c_ between the VR-PS and the PDP RV volume measurements was 0.89 (95% CI 0.781, 0.95). The B&A plots showed good agreement with a mean difference of 1.26 mm^3^ ± 17.31 (95% CI -32.66, 35.19) and a MSE of 284 mm^6^ (Figure 4B).

Comparison of the VR-IBP and VR-PS volume measurements revealed that the VR-IBP measurements were significantly smaller. The LV volumes differed by -18.18 cm^3^ (95% CI -24.63, -11.73), p < 0.001 (t = -5.9, df = 19). The RV volume measurements differed by -8.89 cm^3^ (95% CI -16.97, -0.82), p < 0.05 (t = -2.3, df = 19). Shapiro-Wilk tests indicated that all outcomes were normally distributed.

## 4 Discussion

In the earliest days of 3D anatomical assessment and imaging computation, radiologists used geometric shapes such as planes, spheres, ellipsoids, and truncated cones to define organs and cavities. These structures were used to model the external contours of the human body and the structure of individual organs. These geometrically simple and easy-to-comprehend phantoms were ideal for the primitive computer technology of the time [28, 29, 30]. Though this method was practical, it did not realistically represent the human body anatomically, especially in terms of organ shape and inter-organ tissue separation. As processing speed and memory technology improved during the late 1980s, voxel-based human computational phantoms were developed. These voxel phantoms were created by CMR and CCTA data segmentation using a collection of rectangles of equal or unequal sizes to form anatomical phantoms from unified and standardized elements. This imaging analysis method was based on the tissue shape according to the segmentation and not on approximations, as previously done. The mesh phantom was devised in the 1990s and represents body regions and organ structures by surface curves defined by 3D control points or arrays of polygons rather than voxels. While mesh-type phantoms enable scalability and deformability, they retain the anatomical realism provided by voxel phantoms [31].

**Cardiac function comprehension and 3D anatomical assessments of its chambers:** Simpson's rule has been shown to be an effective tool for estimating cardiac chamber volumes and functions [32, 33, 34]. This method is widely used in daily clinical practice, for different modalities including CMR, CCTA and TTE. Since Simpson's rule is based on the summation of the voxel area of horizontal slices, it is widely accepted to include the papillary muscles and the trabeculae bases (which are topologically variable structures) as part of the intracavitary volume to simplify and standardize the volumetric analysis [35]. This method approximates the LV to a bullet shape, which has been validated clinically and allows for easy evaluation of healthy LV volumes. When pathologically involved, both the RV and the LV present irregular shapes. Thus, using Simpson's rule in such instances may result in volumetric over-estimation or under-estimation [36, 37].

3D VR imaging, unlike the voxel summation method, uses advanced tetrahedron volume summation to estimate chamber volumes. 3D VR is a convenient, fast, and efficient method of assessing complex cardiac structures [38]. Several recent advances in computing technology have made this technology more accessible, thus allowing for reliable spatial perception in real time [39]. This system generates a semi-automatic structure segmentation according to the Hounsfield unit range selected by the operator, thus creating a precise contour and defining only the inner blood pool. In this study, VR volumetric analysis was applied to the heart ventricles and the results compared to the standard volumetric analysis algorithm based on Simpson's rule.

The comparison of the LV volumes calculated by the Phillips dedicated cardiac imaging platform (PDP) and those calculated by the VR-Philips-similar segmentation (VR-PS) showed that in the LV there was an excellent $\rho_{c}$ of 0.982 and the B&A plots also exhibited a good correlation. Thus, Simpson's rule and the polygon summation algorithm produced similar results under the same clinical and technical settings in normal morphological hearts, making this estimation reliable since it was shown to be replicable by different analysis methods. For the RV there was satisfactory agreement between the two methods with a $\rho_{c}$ of 0.89, and the B&A plots showed a good correlation, though not as accurate as in the LV. It was also evident that the semi-automated VR analysis, which relied on Hounsfield units corresponding to the precise LV and RV internal blood pool (VR-IBP), was significantly smaller than the standardized volumes which included the papillary muscles and trabeculae for simplified computation.

Automated 3D segmentation of complex anatomical structures may be inaccurate as a result of low contrast differences, missing edges, and/or low signal-to-noise ratios [40]. Because PDP only considers two tomographic planes while neglecting irregularities, ventricles that differ from bullet-shaped structures may not be well-assessed [41]. Therefore, different congenital heart defects can affect the myocardium, resulting in an abnormally shaped LV. In these cases, fully automated border detection based on Simpson's rule appears to be less reliable than semiautomated methods, which instead of using the voxel summation approximation, can follow the contours of the chambers precisely allowing the ventricular irregularities to be fully evaluated and taken into consideration [42]. These shortcomings of Simpson's rule-based technology could have affected the ability of the PDP to accurately evaluate the RV volumes and may be a plausible explanation for the weaker agreement between the PDP and the VR-PS measurements. Since polygon summation methods have a higher spatial resolution, they thus may be preferable when estimating intricate irregular ventricular volumes in irregular and highly trabeculated left and right ventricles. The significant difference between the VR-IBP and the VR-PS measurements strongly suggests that today's advanced computation abilities allow for easy exclusion of the papillary muscle and the trabeculae, thus facilitating the evaluation of the effective inner blood pool and creating a theoretically more accurate and replicable volumetric analysis of the heart chambers.

## 5 Limitations

This study was performed on a selected population of adult patients’ hearts awaiting left atrial appendage occlusion. Further evaluation needs to be performed on younger patients with anatomically normal, hypertrophic and affected hearts (as seen after myocarditis), and with congenital heart defects in order to establish the superiority of one method over the other. Moreover, it was also assumed the calculation of all the volumes by both modalities were correct, as it was impossible to evaluate the inner blood pool by other techniques.

## 6 Conclusion

Cardiac chamber analysis is currently based on voxel area summation using Simpson's equation. The findings here suggest that polygon summation is a feasible and accurate method for cardiac volume assessment which may be preferable for RV and irregular LV volume and function calculations.

## Declarations

### Author contribution statement

David Yogev and Shai Tejman-Yarden: Conceived and designed the experiments; Performed the experiments; Wrote the paper.

Orly Goitein: Conceived and designed the experiments; Contributed reagents, materials, analysis tools or data; Wrote the paper.

Omer Feinberg: Conceived and designed the experiments; Analyzed and interpreted the data.

Yisrael Parmet: Analyzed and interpreted the data; Wrote the paper.

Shay Ilouz: Performed the experiments; Wrote the paper.

Tomer Goldberg, Netanel Nagar, Dor Freidin, Oliana Vazgovsky, Sumit Chatterji, Yishay Salem and Uriel Katz: Contributed reagents, materials, analysis tools or data; Wrote the paper.

### Funding statement

This research did not receive any specific grant from funding agencies in the public, commercial, or not-for-profit sectors.

### Data availability statement

Data will be made available on request.

### Declaration of interest's statement

The authors declare no conflict of interest.

### Additional information

No additional information is available for this paper.
